# The referral of patients to smoking cessation counselling: perceptions and experiences of healthcare providers in general practice

**DOI:** 10.1186/s12913-021-06618-7

**Published:** 2021-06-17

**Authors:** Naomi A. van Westen-Lagerweij, Elisabeth G. Meeuwsen, Esther A. Croes, Eline Meijer, Niels H. Chavannes, Marc C. Willemsen

**Affiliations:** 1 The Netherlands Expertise Centre for Tobacco Control, Trimbos Institute, PO Box 725, 3500 AS Utrecht, The Netherlands; 2 Department of Health Promotion, Maastricht University, PO Box 616, 6200 MD Maastricht, The Netherlands; 3 Public Health and Primary Care, Leiden University Medical Center, PO Box 9600, 2300 RC Leiden, The Netherlands

## Abstract

**Background:**

Few European smokers receive professional counselling when attempting to quit smoking, resulting in suboptimal success rates and poor health outcomes. Healthcare providers in general practice play an important role in referring smokers to smoking cessation counselling. We chose the Netherlands as a case study to qualitatively explore which factors play a role among healthcare providers in general practice with regard to referral for smoking cessation counselling organised both inside and outside general practice.

**Methods:**

We conducted four focus groups and 18 telephone interviews, with a total of 31 healthcare providers who work in general practice. Qualitative content analysis was used to identify relevant factors related to referral behaviours, and each factor was linked to one of the three main components of the COM-B behaviour model (i.e., capability, opportunity and motivation) as well as the six sub-components of the model.

**Results:**

Dutch healthcare providers in general practice typically refer smokers who want to quit to counselling inside their own general practice without actively discussing other counselling options, indicating a lack of shared decision making. The analysis showed that factors linked to the COM-B main components ‘capability’ and ‘opportunity’, such as healthcare providers’ skills and patients’ preferences, play a role in whether patients are referred to counselling inside general practice. Factors linked to all three COM-B components were found to play a role in referrals to counselling outside general practice. These included (knowledge of) the availability and quality of counselling in the region, patients’ requests, reimbursement, and sense of urgency to refer. The identified factors can both act as barriers and facilitators.

**Conclusions:**

The findings of this research suggest that more smokers can be reached with smoking cessation counselling if implementation interventions focus on: (i) equipping healthcare providers with the knowledge and skills needed to refer patients; (ii) creating more opportunities for healthcare providers to refer patients (e.g., by improving the availability and reimbursement of counselling options); and (iii) motivating healthcare providers to discuss different counselling options with patients.

**Supplementary Information:**

The online version contains supplementary material available at 10.1186/s12913-021-06618-7.

## Background

Tobacco use remains a major public health issue, especially in Europe where it is estimated that 29 % of citizens above 15 years old use tobacco products [1]. As many European countries strive towards becoming tobacco-free, ensuring that smokers have access to evidence-based cessation methods is becoming increasingly important. The most effective cessation method is a combination of pharmacotherapy and intensive behavioural counselling, the latter provided either face-to-face (individually or in a group) or via telephone [2].

Currently, use of evidence-based cessation methods is low across European countries [3–5], and even declined between 2012 and 2017 [6]. As such, despite the availability of many types of evidence-based interventions, the impact on public health remains low. General practice is an important source for smokers to access evidence-based cessation care [7]. National guidelines in many countries recommend primary healthcare providers to ask patients whether they smoke, to advise smokers to quit smoking, and to offer behavioural counselling and pharmacological support to smokers who want to quit smoking [8]. Healthcare providers (HCPs) in general practice typically refer patients to smoking cessation counselling (SCC) organised either inside or outside general practice, depending on a country’s smoking cessation infrastructure. The referral behaviour of HCPs in general practice may, however, be influenced by various factors such as patient reimbursement, the awareness and knowledge of (the quality of) local smoking cessation services, and patients’ and HCPs’ attitudes towards SCC [9–13].

In order to reach more smokers with SCC, it is important to know which factors are related to the referral behaviour of HCPs in general practice. According to the COM-B behaviour model, behaviour (B) is generated by three components: capability (C), opportunity (O), and motivation (M) [14]. Capability refers to the knowledge and skills which are necessary to perform a certain behaviour; opportunity refers to the external factors which make a certain behaviour possible; motivation refers to internal processes such as decision making and emotions which influence behaviour [14]. Each component can be further divided into two sub-components. With regard to capability, one can distinguish between physical capability (e.g., physical strength and skills) and psychological capability (e.g., knowledge, comprehension and reasoning). Opportunity comprises physical opportunity (i.e., opportunity afforded by the environment, such as time and location) and social opportunity (i.e., opportunity which is a result of social factors, such as cultural norms). Motivation involves reflective processes (e.g., making plans and evaluations) and automatic processes (e.g., emotions, desires and impulses) [14]. The COM-B behaviour model has successfully been used in other studies aimed at improving the behaviour of HCPs involved in smoking cessation care [15, 16]. By using this model, one can identify which components play a role in the referral behaviour of HCPs in general practice and thus select appropriate behaviour change interventions [14].

Within Europe, the Netherlands is an interesting case to examine, as SCC is organised both inside and outside the general practice setting. Most Dutch general practices have a practice nurse (PN) whose main task is to provide chronic disease care, including counselling smokers [17, 18]. As a result, smokers are usually referred by the GP to the PN. Two types of PNs exist within general practice: PNs who are specialised in somatic care and PNs who are specialised in mental health care [17, 18]. Typically, SCC is provided by a PN who is specialised in somatic care [18].

Also, many options for SCC exist outside general practice, which may be especially useful for general practices faced with a high workload (e.g., due to COVID-19) or a lack of expertise to counsel patients. Commercial organisations, self-employed coaches and smoking cessation outpatient clinics are examples of options outside general practice which patients can be referred to [19, 20]. While counselling inside general practice typically involves individual and/or telephone counselling, counsellors outside general practice often also offer group counselling and/or more specialised care for particular subgroups (such as pregnant women and heavily addicted smokers) [20]. Some of these counsellors require an official referral letter by the general practitioner (GP) [20], indicating the central role of the GP as gatekeeper to SCC.

Only SCC provided by qualified counsellors (i.e., counsellors registered in the national Quit Smoking Quality Register) is reimbursed by healthcare insurance companies once a year; this includes SCC both inside and outside general practice [19]. Despite the many possibilities for SCC in the Netherlands, no more than 5 % of smokers who make a serious quit attempt (i.e., refraining from smoking for at least 24 h) currently receive professional counselling provided either inside or outside general practice [21], which may partly be explained by the fact that Dutch GPs often prescribe cessation medication without behavioural counselling [22]. The aim of this study was to qualitatively explore, from the perspective of Dutch HCPs in general practice, which factors play a role in the referral of smokers to SCC organised both inside and outside general practice.

## Methods

### Study design and participants

This qualitative study was based on the answers of 31 HCPs who work in general practice. We conducted four semi-structured focus groups on smoking cessation care in general, followed by 18 semi-structured individual telephone interviews on referrals to SCC specifically. The focus groups were part of a larger study, aimed at developing a new referral strategy to ensure that more smokers are referred to behavioural counselling (the focus groups were presented to HCPs within this context). While the interviews were also part of this study, they were solely focused on exploring the experiences of HCPs regarding the referral of patients and not aimed at developing a new referral strategy.

For the focus groups, we recruited HCPs from both primary and secondary care. Participants were not required to be actively involved in smoking cessation care. Different recruitment channels were employed: newsletters sent out through professional associations, e-mails sent directly to practices in the regions of the research institutes, e-mails sent directly to HCPs registered in the Quit Smoking Quality Register, and e-mails sent directly to HCPs who participated in an earlier study on implementation of smoking cessation care [9]. We aimed for a minimum of five and maximum of eight participants per focus group, as recommended in the literature [23]. We recruited 22 HCPs; however, due to three last-minute withdrawals we included 19 HCPs (five participants in three focus groups and four participants in one focus group). Thirteen HCPs worked in general practice at the time, of which three GPs, seven PNs who are specialised in somatic care, two doctor’s assistants (DAs), and one pulmonary nurse. For the purpose of this study, only the results of these 13 HCPs will be reported.

Considering the small number of GPs that participated in the focus groups compared to the other professions, we decided to only conduct additional telephone interviews with GPs. A total of 18 GPs were recruited through our professional network as well as e-mails sent directly to HCPs who participated in an earlier study on smoking cessation [9].

### Procedure

The focus groups were conducted in May and June 2019 in the cities of Utrecht and Leiden. The telephone interviews were conducted in February and March 2020, just before COVID-19 impacted healthcare in the Netherlands. All participants received written information about the study before participation and were informed about the purpose of the study and confidentiality procedures. Participants were informed that participation is voluntary and that participation may be discontinued at any time. The travel expenses of the focus group participants were reimbursed.

The focus groups were led by the first and second author. The first author moderated two focus groups, while the second author made field notes, and vice versa. Both authors were doctoral researchers with a background in health policy (first author) and medicine (second author), and with experience in conducting qualitative research; they had no relationship with the participants prior to study commencement. Before the start of the focus groups, participants were asked to sign an informed consent form. The telephone interviews were conducted by the first author. Interview participants received the informed consent form beforehand through e-mail and provided verbal informed consent which was recorded at the start of the interview, as approved by the Trimbos Institutional Ethics committee.

Semi-structured focus group and interview guides were used to guide the conversations (see Additional File 1 for the questions). In the focus groups, participants were asked to share their experiences with smoking cessation care and their views on how to improve smoking cessation care in the Netherlands. The ‘referral of patients to SCC’ was one of the discussed topics. In the interviews, participants were asked why they do or do not refer patients to SCC, and which factors (would) make it easier for them to refer patients to SCC.

The focus groups lasted between 83 and 96 min (90 min on average) and the telephone interviews lasted between 11 and 23 min (15 min on average). The focus groups and telephone interviews were recorded and transcribed verbatim. Quotes presented in this article were translated from Dutch to English by the first author.

### Ethics

 This study was conducted according to the guidelines of the Helsinki Declaration of Good Clinical Research Practice and was approved by the Trimbos Institutional Ethics committee.

### Analysis

Qualitative content analysis was conducted after all focus groups and interviews were completed and transcribed, using the software package ATLAS.ti. The first and second author independently coded one randomly selected focus group transcript and two randomly selected interview transcripts, using the topics of the focus group and interview guides (thematic coding). In addition, they applied open coding to capture relevant data. Through discussing their codes, the authors resolved discrepancies in coding and agreed upon new codes and categories (axial coding). The first author coded the remaining transcripts. New codes that emerged were discussed between the two authors (see Supplementary Tables 1, Additional File 2 for the final codes). Theme saturation was established following analysis of the four focus groups and the first 13 conducted interviews, meaning that analysis of the remaining five interviews did not lead to any new emergent themes.

For the purpose of this study, only the final codes of the HCPs who work in general practice were used to identify factors related to referrals. This included the codes from all 18 interview participants, as well as the 13 focus group participants who work in general practice. Using the final codes, the first author made two overviews: firstly of factors related to in-practice referrals (i.e., referrals to the PN), and secondly of factors related to referrals to counselling outside general practice. Only factors mentioned by at least two HCPs were included, as factors mentioned by just one HCP were considered to be unique to the individual and thus likely not applicable to other HCPs. The identified factors were continuously compared against the transcripts and adjusted if necessary. The first and second author then independently linked each factor to one of the three main components as well as the six sub-components of the COM-B behaviour model and resolved most of their discrepancies. Any remaining discrepancies were resolved with the help of the fourth author.

## Results

Table 1 presents the characteristics of the participants. Thirty-one HCPs who work in general practice participated, of which 12 were male (39 %). The mean age was 51 years (SD 9), the mean professional experience was 16 years (SD 10) and none of the participants smoked. About half of the participants (52 %) worked at a practice situated in a large urban area (i.e., a municipality with 1500 or more housing units per square kilometre). All participants indicated that they often ask patients whether they smoke, especially if there is a smoking-related health problem, and usually provide a quit advice to those who smoke.

**Table 1:** Characteristics of the participants

*Characteristics*	*n = 31*
**Gender – n (%)**	
Male	12 (39)
Female	19 (61)
**Age (in years)**	
Mean (SD)	51 (9)
**Profession – n (%)**	
General practitioner	21 (68)
Practice nurse	7 (23)
Doctor’s assistant	2 (6)
Pulmonary nurse	1 (3)
**Professional experience (in years)**	
Mean (SD)	16 (10)
**Smoking status – n (%)**	
Non-smoker	31 (100)
Smoker	0 (0)
**Practice location – n (%)**	
Large urban area (1500 or more housing units per km^2^)	16 (52)
Small urban or suburban area (1000 to 1500 housing units per km^2^)	3 (9)
Rural area (fewer than 1000 housing units per km^2^)	12 (39)

### SCC organised within general practice

 Twenty-nine (out of 31) participants mentioned that patients who want to quit are usually offered individual face-to-face or telephone counselling within their practice. Most participants indicated that they only discuss other types of counselling if patients actively inquire about other options or when counselling within practice is not sufficient (e.g., due to multiple addictions).

The participating PNs, one DA and the pulmonary nurse are all qualified to deliver SCC in their practice. In the practices of the other participating DA and 18 (out of 21) GPs, a qualified PN specialised in somatic care delivers SCC. Seven HCPs mentioned that they sometimes refer patients to their PN specialised in mental health care, mainly when patients experience psychological or psychosocial barriers in quitting.

Three participating GPs mentioned that they do not have a qualified PN in their practice. As a result, two of these GPs always refer to counselling outside the practice; despite the lack of qualification the third GP still offers counselling inside the practice, which is not reimbursed.

 Overall, participants were satisfied with the counselling offered in their practice, although they were less positive about the low financial compensation they received for it. Several participants also mentioned that they experience difficulty in counselling certain groups of patients, especially those who are severely addicted to smoking.

*‘I personally have a need for sending patients to addiction care when smoking is really persistent. You can go there for an alcohol or drug addiction, but for some reason smoking is hardly treated there, while quitting smoking is not necessarily easier than quitting alcohol or drugs. (…) I notice that we sometimes get stuck and I think that is a shame. Perhaps those patients could get further with [addiction care].’ (P17, GP)*.

While patients are typically referred to the PN for counselling, we found that this is not always the case: sometimes GPs decide to offer patients medication and/or behavioural counselling themselves, without referring to the PN. We identified four factors related to referrals inside general practice, presented in Supplementary Table 2 (in Additional File 3). We linked one factor to the COM-B sub-component ‘psychological capability’, one factor to ‘social opportunity’ and two factors to ‘physical opportunity’.

Most GPs mentioned that they leave the responsibility with their patients to plan an appointment for SCC.

*‘[To patients who smoke] I say: “know that the door is open”, but I let them take the next step. So when I ask them if they want to quit smoking, and they say “yes I want to”, then they need to take the next step to plan an appointment with me or the practice nurse.’ (P19, GP)*.

Several focus group participants remarked that leaving the responsibility with patients often resulted in no-shows. These same participants experienced it works better if HCPs are more directive and take the responsibility to plan a follow-up appointment for their patients.

*‘For lifestyle issues, the GP now asks patients if they are interested to see me, after which I will call the patients to make an appointment. (…) Before, when they had to make an appointment themselves, they often didn’t show up.’ (P11, DA)*.

### SCC organised outside general practice

 Regarding counselling organised outside general practice, 16 participants mentioned they occasionally refer patients to a commercial organisation (mostly group counselling; two HCPs also referred patients to telephone counselling), an addiction care specialist, or a specialist at the hospital. Referrals to counselling outside general practice are mostly made upon patients’ request. All but one of the participants never refer patients to a self-employed coach. Most participants mentioned being open to the idea of referring patients outside general practice, especially to group counselling and addiction care.

Supplementary Table 3 (in Additional File 4) shows an overview of all the factors related to referrals outside general practice (n = 20), each linked to a COM-B sub-component. We identified three factors linked to the sub-component ‘psychological capability’; seven factors linked to ‘physical opportunity’; two factors linked to ‘social opportunity’; five factors linked to ‘reflective motivation’; and three factors linked to ‘automatic motivation’. The six most mentioned factors were: (1) knowledge of counselling in the region (psychological capability), (2) the actual availability of counselling in the region (physical opportunity), (3) requests from patients to be referred to counselling outside general practice (social opportunity), (4) reimbursement of counselling (physical opportunity), (5) perceptions of the quality of counselling outside general practice (reflective motivation), (6) sense of urgency to refer patients to counselling outside general practice (reflective motivation).

Important to note is that several identified factors can both act as barriers and facilitators (in Supplementary Table 3 these factors are supported by two quotes). For example, regarding the availability of counselling in the neighbourhood, the absence or lack of counselling in the neighbourhood inhibits referrals, while the presence of counselling in the neighbourhood stimulates HCPs to refer. Overall, barriers were more often mentioned than facilitators.

## Discussion

### Main findings

This study identified a multitude of factors which play a role in the referral behaviour of HCPs. Regarding in-practice referrals, factors linked to the COM-B main components ‘capability’ and ‘opportunity’ played a role; regarding referrals to counselling outside general practice, factors linked to all three COM-B main components (capability, opportunity and motivation) were found to be relevant.

### Interpretation of findings

Our results seem to be consistent with previous research which suggested that the referral behaviour of HCPs in general practice is associated with patient reimbursement, collaboration agreements between primary HCPs, the awareness and knowledge of (the quality of) local smoking cessation services, and patients’ and HCPs’ attitudes towards SCC [9–13]. Using the Netherlands as a case study, we propose new factors that may play a role in the referral behaviour of HCPs in general practice, such as the actual availability of counselling in the region, requests from patients, personally knowing counsellors, a sense of urgency to refer, and HCPs’ own (perception of) skills and abilities in counselling and referring patients. These factors, especially those related to counselling outside general practice, likely also play a role in other countries where SCC is mostly provided outside general practice.

We found that quite a number of factors were related to whether HCPs refer to counselling outside general practice, while only a few factors were mentioned in relation to in-practice referrals, underlining the importance of distinguishing between these two types of referrals. Moreover, motivational factors appear to play a role in referrals to counselling outside general practice, but not in-practice referrals. A possible explanation is that in-practice referrals take place between two HCPs who already know and trust each other, while referrals to counselling outside general practice are usually made to an unknown counsellor, and therefore factors such as the quality and trustworthiness of the counsellor are considered before such a referral is made.

In addition, most HCPs work in a practice with a PN whom they can refer patients to for individual face-to-face or telephone counselling and whom they are usually satisfied with, thus lowering their need for counselling outside general practice. This may also explain why HCPs appear to be more open towards referring patient to group counselling and addiction care: since PNs typically do not offer group counselling and addiction care within practice, HCPs may feel a greater sense of urgency to refer to these types of counselling.

A notable finding is that the referral behaviour of HCPs appears to be strongly related to their perceptions of what patients do or do not want. These perceptions seem to be partly based on experience: for example, smokers may express negative attitudes towards counselling [24, 25] and may only want medication. On the other hand, HCPs’ perceptions also seem to be partly based on assumptions: when patients do not actively request to be referred to a specific type of counselling, many HCPs directly refer to the PN without discussing alternative options. However, an overlooked reason why smokers may not ask for a specific type of counselling, is because they may not be aware of its availability [24, 26]. Research suggests that many smokers will accept smoking cessation support if it is actively offered by HCPs [27]. A culture shift is needed in which HCPs actively discuss all options for counselling and explain the benefits of each option, and/or provide a decision aid, to help smokers make an informed choice while stimulating a more positive attitude towards counselling. Ultimately, this will increase decisional quality, patient satisfaction and quit attempts [28–30].

### Implications

Our findings provide a basis for developing and implementing interventions to ensure that more smokers receive behavioural counselling [14]. First, our findings show that Dutch HCPs who work in general practice typically refer smokers who want to quit to individual or telephone counselling within general practice and hardly actively discuss other options for SCC. As a result, patients may not receive the type of SCC which is best suited to their needs. We, therefore, propose that HCPs should be educated about the importance of shared decision making. Moreover, HCPs who prefer to keep patients within their own practice should be encouraged to offer different types of SCC within their practice, for example by working together with a counsellor who can provide a different type of SCC within the practice.

Second, barriers may exist which make referring difficult or even impossible, resulting in less smokers being reached with SCC. For example, an important barrier is the lack of (knowledge of) referral options for HCPs who want to refer their patients to SCC outside general practice. Primary care organisations may play a role in creating more referral options and informing HCPs about the availability and quality of different options. As a result of the current limitations in face-to-face counselling due to COVID-19, more counsellors are now providing their services at a distance (e.g., through video calls or by telephone). Counselling at a distance may also be a solution for HCPs who work in a region where counselling is hardly available.

Another frequently encountered barrier is the preference of patients to quit without counselling, thus making it impossible for HCPs to refer patients. As long as patients are not convinced of the added value of SCC, the public health impact of improving the referral system will remain limited. Therefore, providing HCPs with training on how to convince patients of the added value of SCC, as well as launching mass media campaigns to inform smokers about the importance and benefits of counselling, may be necessary to improve the utilisation of SCC among smokers.

On a final note, the effect of all abovementioned interventions will remain limited if referral options are not reimbursed by healthcare insurance companies. It is, therefore, imperative that countries implement policies which ensure full reimbursement of SCC by healthcare insurance companies.

### Strengths and limitations

To our knowledge, this study was the first exploration of the factors related to referrals to different types of SCC from the perspective of HCPs in general practice. The generalisability of the results is, however, subject to some limitations. First, our results are based on the Dutch smoking cessation infrastructure in which the PN holds a unique position, and may therefore be less applicable to other countries. Nonetheless, as mentioned earlier, some of the challenges presented in this paper likely apply to other countries as well. Researchers interested in studying this topic and comparing countries may find it useful to replicate our approach. Also, countries that wish to adopt a SCC system similar to the Dutch system may use the insights from this study.

Second, our sample of participants mainly included HCPs who are actively involved in smoking cessation care and who do not smoke. Quantitative research may inform whether the identified factors are representative of the larger population of HCPs.

 Third, the conversations tended to focus on referrals to counselling outside general practice, since participants were mostly positive about the counselling offered in their own practice. This means we may have missed some factors related to in-practice referrals. Also, we decided to use the COM-B behaviour model in the analysis after the data was collected. This means our questions in the focus group and interviews did not specifically address the three main components of the model, and we may thus have missed some factors. On the other hand, not having a theoretical model at the beginning of the research allowed for an inductive approach during the analysis in which factors were identified which otherwise would have been missed.

Fourth, we included two data collection methods (focus groups and interviews) which both had different sets of questions. This may partly explain why we found several additional factors in the interviews which were not mentioned in the focus groups. Nevertheless, the use of both focus groups and interviews contributed to a more comprehensive understanding of the referral behaviour of HCPs, since we would have missed the additional factors if we had only analysed the focus groups.

Finally, the interviews were conducted a year after the focus groups, which means that societal developments may have impacted some of the findings. As of 2020, more smoking cessation programmes are fully reimbursed, meaning that SCC has become more accessible to patients. Most participants, however, mentioned that they had not noticed any differences in the requests of patients, which shows that the development probably had a minor effect on our results.

## Conclusions

The present research identified several new factors which play a role in the referral behaviour of HCPs in general practice with regard to SCC. The findings of this research suggest that more patients can be reached with SCC if implementation interventions focus on equipping HCPs’ with the knowledge and skills needed to refer patients; creating more opportunities for HCPs to refer patients; and motivating HCPs to discuss different SCC options with patients.

## Supplementary Information

**Additional file 1.** Focus group and interview questions.

**Additional file 2.** Final codes.

**Additional file 3.** Factors related to referrals inside general practice.

**Additional file 4.** Factors related to referrals outside general practice.

## Data Availability

The datasets used and/or analysed during the current study are available from the corresponding author on reasonable request.

## Abbreviations

HCP: Healthcare provider
SCC: Smoking cessation counselling
GP: General practitioner
PN: Practice nurse
DA: Doctor’s assistant
