# Supplementary material for: The referral of patients to smoking cessation counselling: perceptions and experiences of healthcare providers in general practice
Source: BMC Health Serv Res. 2021 Jun 17;21:583. doi: 10.1186/s12913-021-06618-7 (PMC8210508; doi:10.1186/s12913-021-06618-7)
Supplement: Supplementary file 1 — Additional file 1. Focus group and interview questions. [file 12913_2021_6618_MOESM1_ESM.docx]

Additional File 1

**Title:** The Referral of Patients to Smoking Cessation Counselling: Perceptions and Experiences of Healthcare Providers in General Practice

**Authors**: Naomi A. van Westen-Lagerweij, Elisabeth G. Meeuwsen, Esther A. Croes, Eline Meijer, Niels H. Chavannes, Marc C. Willemsen

**Focus group questions**

*Only the questions relevant for this study are presented here. Demographic questions were asked in a separate questionnaire.*

1) What is your profession and to what extent are you involved in smoking cessation care?

2) Who is responsible for smoking cessation care in your general practice?

3) Do you refer patients to other healthcare providers for smoking cessation care?

- If yes: What is the referral process like? What are areas for improvement? What are best practices?

4) Are patients referred to you by other healthcare providers for smoking cessation care?

- If yes: What is the referral process like? What are areas for improvement? What are best practices?

5) What are your thoughts on the organisation of smoking cessation care in the Netherlands? What are areas for improvement? What are best practices?

**Interview questions**

1) What is your age?

2) How long have you been working as a general practitioner?

3) Do you smoke?

4) What percentage of your patients do you think smokes?

5) What is the most common socioeconomic status among your patients (high/middle/low)?

6) Recently, smoking cessation has received much attention. Have you noticed any change among your patients?

7) How important is the subject smoking cessation for you on a scale from 1 to 10, and why?

8) How is smoking cessation care organised in your practice?

9) What is your role in smoking cessation care in your practice?

- What are your actions with regard to patients who smoke and why/when do you take these actions?

- What kind of support do you offer?

10) Have you been trained to provide smoking cessation care?

11) Do you refer smokers to behavioural counselling?

- If yes: What kind of behavioural counselling do you refer to and why? Who offers that kind of counselling? What is the referral process like? What are you satisfied or dissatisfied with?

- If no: Why not? (Or: why do you not refer to a certain type of counselling?)

12) Do you refer patients to alternative therapy?

*Additional questions that may be asked if they haven’t come up yet:*

13) What makes referring easy for you as a general practitioner?

14) What are your thoughts on referring patients to counselling outside general practice?

15) What are your thoughts on the availability of counselling in the region?
