# Supplementary material for: The referral of patients to smoking cessation counselling: perceptions and experiences of healthcare providers in general practice
Source: BMC Health Serv Res. 2021 Jun 17;21:583. doi: 10.1186/s12913-021-06618-7 (PMC8210508; doi:10.1186/s12913-021-06618-7)
Supplement: Supplementary file 2 — Additional file 2. Final codes. [file 12913_2021_6618_MOESM2_ESM.docx]

Additional File 2

**Title:** The Referral of Patients to Smoking Cessation Counselling: Perceptions and Experiences of Healthcare Providers in General Practice

**Authors:** Naomi A. van Westen-Lagerweij, Elisabeth G. Meeuwsen, Esther A. Croes, Eline Meijer, Niels H. Chavannes, Marc C. Willemsen

**Supplementary Table 1.** Final codes.

| **Category** | **Codes** |
| --- | --- |
| 1. HCP characteristics | 1.1 Experience  1.2 Training  1.3 Importance smoking cessation  1.4 Smoking history |
| 2. Current smoking cessation care in practice | 2.1 Patient population  2.1.1 Percentage smokers  2.1.2 Socioeconomic status  2.2 Organisation smoking cessation care  2.2.1 Type of counselling  2.3 Addressing the subject  2.4 Advising to quit  2.5 Discussing options  2.5.1 Responsibility patient  2.6 Treating patients  2.6.1 Prescribing medication  2.7 Referring patients  2.7.1 In-practice referrals  2.7.1 External referrals  2.8 Receiving referrals  2.9 Alternative therapy  2.10 Best practices |
| 3. Factors related to referrals | 3.1 Availability in region  3.1.1 Knowledge of availability  3.1.2 Contact with counsellors  3.1.3 Group counselling  3.2 Reimbursement  3.2.1 Health insurers  3.2.2 Addiction care  3.3 HCP factors  3.3.1 Added value  3.3.2 Perceptions of counsellors  3.4 Patient factors  3.5 Referral system  3.6 Other |
| 4. Role of HCPs | 4.1 Role GP  4.2 Role PN  4.3 Role DA  4.5 Role other primary HCPs  4.6 Role healthcare organisations  4.7 Role external counsellors |
