# Supplementary material for: The referral of patients to smoking cessation counselling: perceptions and experiences of healthcare providers in general practice
Source: BMC Health Serv Res. 2021 Jun 17;21:583. doi: 10.1186/s12913-021-06618-7 (PMC8210508; doi:10.1186/s12913-021-06618-7)
Supplement: Supplementary file 3 — Additional file 3. Factors related to referrals inside general practice. [file 12913_2021_6618_MOESM3_ESM.docx]

Additional File 3

**Title:** The Referral of Patients to Smoking Cessation Counselling: Perceptions and Experiences of Healthcare Providers in General Practice

**Authors:** Naomi A. van Westen-Lagerweij, Elisabeth G. Meeuwsen, Esther A. Croes, Eline Meijer, Niels H. Chavannes, Marc C. Willemsen

**Supplementary Table 2.** Factors related to referrals inside general practice.

| **COM-B main component** | **Factor** | **COM-B sub-component** | **Examples of quotes** | **Mentioned by** | **Source(s)** |
| --- | --- | --- | --- | --- | --- |
| Capability | HCPs’ skills | Psychological capability | ‘I'm not very good at conversation techniques and behavioural change, but I am good at delegating, so I like to delegate [those tasks] to the PN.’ (P24, GP) | 6 GPs, 1 DA | Focus groups & interviews |
| Opportunity | Patients’ preferences, e.g. some patients only want medication, or only want to be treated by the GP | Social opportunity | ‘[There are] people who don't have time for [counselling], who immediately say 'I want Champix'. (…) I give them a prescription because they don't want to be referred [to the PN].’ (P14, GP)  ‘There are some people who do not like going to a PN because they feel it is better to stay with the GP.’ (P29, GP) | 7 GPs | Focus groups & interviews |
|  | HCP’s time for counselling | Physical opportunity | ‘I always provide counselling, because I have a lot more time for it. The GP does not have time for that.’ (P6, PN)  ‘I refer 9 out of 10 [patients] to our PN, who then starts the smoking cessation process with them. However, I treat some people myself, especially when I see an opportunity at that moment and I don’t want to have a delay. ‘ (P1, GP) | 5 GPs, 2 PNs | Focus groups & interviews |
|  | Capacity in the practice | Physical opportunity | ‘We have 4 PNs in our practice who can all provide counselling. However, two of them are ill at the moment, so I now counsel one patient myself.’ (P30, GP) | 1 GP | Interviews |
| Motivation | - | - | - | - | - |
