# Supplementary material for: The referral of patients to smoking cessation counselling: perceptions and experiences of healthcare providers in general practice
Source: BMC Health Serv Res. 2021 Jun 17;21:583. doi: 10.1186/s12913-021-06618-7 (PMC8210508; doi:10.1186/s12913-021-06618-7)
Supplement: Supplementary file 4 — Additional file 4. Factors related to referrals outside general practice. [file 12913_2021_6618_MOESM4_ESM.docx]

Additional File 4

**Title:** The Referral of Patients to Smoking Cessation Counselling: Perceptions and Experiences of Healthcare Providers in General Practice

**Authors:** Naomi A. van Westen-Lagerweij, Elisabeth G. Meeuwsen, Esther A. Croes, Eline Meijer, Niels H. Chavannes, Marc C. Willemsen

**Supplementary Table 3.** Factors related to referrals outside general practice.

| **COM-B main component** | **Factor** | **COM-B sub-component** | **Examples of quotes** | **Mentioned by** | **Source(s)** |
| --- | --- | --- | --- | --- | --- |
| Capability | Knowledge of counselling in the region, especially group counselling | Psychological capability | ‘I think [group counselling is offered] at the hospital, but I’m not sure. No, group counselling is actually quite unknown to me.’ (P23, GP)  “I don’t know anything [about the availability of external counselling. If I did], I would definitely refer [patients].” (P14, GP) | 11 GPs, 2 PNs | Focus groups & interviews |
|  | Ability to convince/ motivate patients to go to counselling outside general practice | Psychological capability | ‘If I were to mention [counselling outside the practice], I think there would be a few patients who would say ‘doctor, I will do that.’ (…) If I were to encourage that, I think I would be able to only motivate a few patients.’ (P26, GP) | 2 GPs | Interviews |
|  | Ability to successfully help patients to quit within practice | Psychological capability | ‘We refer patients to group counselling when we notice that the individual counselling by our PN doesn’t work well.’ (P20, GP) | 1 GP, 1 PN | Focus groups & interviews |
| Opportunity | The actual availability of counselling in the region | Physical opportunity | ‘There really is a lack of group counselling. My patients regularly say: ‘I would like to do something in a group, to share experiences.’ I just can’t find out where that is.’ (P12, DA)  ‘There is no [addiction care] in the neighbourhood. (…) [Otherwise] I would definitely [refer patients there].’ (P14, GP) | 9 GPs, 2 PNs, 1 DA | Focus groups & interviews |
|  | Requests from patients to be referred to counselling outside general practice | Social opportunity | ‘The main reason I don’t mention [counselling outside general practice] is because patients don’t ask for it.’ (P26, GP)  ‘Each year there are a couple of people who say ‘I want to go to the smoking cessation outpatient clinic’. (…) They already looked into it beforehand. I don’t want to argue with them.’ (P18, GP) | 9 GPs | Interviews |
|  | Reimbursement of counselling | Physical opportunity | ‘What I especially want is an offer for people who are severely addicted to smoking. (…) You can’t send someone to [an addiction care specialist] for a nicotine addiction [only].’ (P15, GP)  ‘[I] sometimes [refer to counselling outside general practice], especially when patients want to have it reimbursed.’ (P21, GP) | 6 GPs, 2 PNs | Focus groups & interviews |
|  | Patient barriers, especially towards group counselling | Social opportunity | ‘When you offer group counselling to people, they say ‘that might be good for my neighbours, but not for me.’ I think the threshold is very high.’ (P30, GP) | 5 GPs, 1 PN | Focus groups & interviews |
|  | The location of counselling | Physical opportunity | ‘[The distance to group counselling] is 12 or 15 kilometres. For some people, that's just too much to bridge when they don't have transportation.’ (P20, GP)  ‘I think it’s good if [group counselling] is neighbourhood-oriented, meaning it’s present in the neighbourhood of the patient and patients can easily contact them.’ (P24, GP) | 3 GPs | Interviews |
|  | Referral system | Physical opportunity | ‘Referring to a group should be: I know where a group is and [patients] can sign up there, and there’s no administrative hassle. (…) The referral system should be really simple.’ (P24, GP) | 2 GPs, 1 PN | Focus groups & interviews |
|  | Stability of external counsellors | Physical opportunity | ‘I find it a bit inconvenient that [counsellors] are here for a while and there for a while (…) and then they leave again. I think that if they were a bit bigger, they would be more stable.’ (P21, GP) | 1 GP | Interviews |
|  | Time to look into referral options | Physical opportunity | ‘I have never looked into [referral options] before. But that is really because I am not a very motivated GP anymore, and the practice I work at (…) is so crazy busy. I’m stressed out all the time.’ (P14, GP) | 1 GP | Interviews |
|  | Availability of counselling outside working hours for patients who don’t have time during the day | Physical opportunity | ‘If [patients] are not able to go [to our PN] during the day, we refer them to [another] general practice for group counselling.’ (P11, DA) | 1 DA | Focus groups |
| Motivation | Sense of urgency to refer patients to counselling outside general practice | Reflective motivation | ‘No I don’t feel the urge [to refer]. If people want to quit smoking and we can offer help and they think it’s fine, then I’m okay with that.’ (P24, GP)  ‘Look, my PN does a great job but (…) smoking cessation is so important that we have to reach a much larger group [of patients].’ (P27, GP) | 7 GPs | Interviews |
|  | Perceptions of the quality of counselling outside general practice | Reflective motivation | ‘The problem with self-employed counsellors is: how do you know if someone delivers quality work? (…) If I refer a patient, then I actually want to know if that counsellor is a good one.’ (P3, GP) | 5 GPs, 1 PN, 1 DA | Focus groups & interviews |
|  | Preference to keep patients within the practice | Reflective motivation | ‘I try to keep [patients] within my own practice, because I then (…) know what happens and can see and monitor them myself.’ (P25, GP) | 6 GPs | Interviews |
|  | Personally knowing and trusting a counsellor | Automatic motivation | ‘You can have a social map, but if you don’t actually know anybody, then you won’t refer either.’ (P10, PN)  ‘I only send people to [counselling outside general practice] when I know who [the counsellor] is, and when I trust [the person]. (…) [It helps] when I have met the person first.’ (P15, pulmonary nurse) | 3 GPs, 1 PN, 1 pulmonary nurse | Focus groups & interviews |
|  | Resistance towards commercial counsellors | Automatic motivation | ‘I have some resistance towards a commercial party which the patient has to pay for. (…) Commercial coaches need to get money from somewhere, so they treat patients from a commercial point of view, while I as a GP have no commercial interest in someone who quits smoking. ‘ (P19, GP) | 2 GPs | Interviews |
|  | Motivation to look into referral options | Automatic motivation | ‘I have never looked into [referral options] before. But that is really because I am not a very motivated GP anymore, and the practice I work at (…) is so crazy busy. I’m stressed out all the time.’ (P14, GP) | 1 GP | Interviews |
|  | Not wanting to argue with patients | Reflective motivation | ‘Each year there are a couple of people who say ‘I want to go to the smoking cessation outpatient clinic’. (…) They already looked into it beforehand. I don’t want to argue with them.’ (P18, GP) | 1 GP | Interviews |
|  | Not wanting to miss out on income | Reflective motivation | ‘I organized [group counselling] for the whole region, and I had to deal with resistance from colleagues from other practices. They said: “now I am going to miss out on income.”’ (P27, GP) | 1 GP | Interviews |
